# Gene regulatory networks controlled by FLOWERING LOCUS C that confer variation in seasonal flowering and life history

**DOI:** 10.1093/jxb/eraa216

**Published:** 2020-05-05

**Authors:** Eva Madrid, John W Chandler, George Coupland

**Affiliations:** 1 Max Planck Institute for Plant Breeding Research, Carl-von-Linné-Weg, Germany; 2 University of Cologne, Germany

**Keywords:** Floral transition, FLOWERING LOCUS C, *FT*, MADS-domain, *SQUAMOSA PROMOTER BINDING PROTEIN-LIKE 15*, *SUPPRESSOR OF OVEREXPRESSION OF CONSTANS 1*, vernalization

## Abstract

Responses to environmental cues synchronize reproduction of higher plants to the changing seasons. The genetic basis of these responses has been intensively studied in the Brassicaceae. The MADS-domain transcription factor FLOWERING LOCUS C (FLC) plays a central role in the regulatory network that controls flowering of *Arabidopsis thaliana* in response to seasonal cues. FLC blocks flowering until its transcription is stably repressed by extended exposure to low temperatures in autumn or winter and, therefore, FLC activity is assumed to limit flowering to spring. Recent reviews describe the complex epigenetic mechanisms responsible for *FLC* repression in cold. We focus on the gene regulatory networks controlled by FLC and how they influence floral transition. Genome-wide approaches determined the *in vivo* target genes of FLC and identified those whose transcription changes during vernalization or in *flc* mutants. We describe how studying FLC targets such as *FLOWERING LOCUS T*, *SQUAMOSA PROMOTER BINDING PROTEIN-LIKE 15*, and *TARGET OF FLC AND SVP 1* can explain different flowering behaviours in response to vernalization and other environmental cues, and help define mechanisms by which FLC represses gene transcription. Elucidating the gene regulatory networks controlled by FLC provides access to the developmental and physiological mechanisms that regulate floral transition.

## Teaser

We describe how genes repressed by FLOWERING LOCUS C contribute to the regulatory network that controls flowering in response to environmental cues and developmental age in annual and perennial Brassicaceae.

## Introduction

Many plant developmental programmes are responsive to environmental cues. This is particularly evident in the characteristic seasonal patterns of flowering. These patterns have adaptive value in synchronizing reproduction with appropriate environmental conditions, maximizing the number of progeny produced, and thereby contributing to fitness in natural environments or yield in agriculture. In temperate climates, winter temperatures (vernalization) and daylength (photoperiod) provide two major floral induction cues (Andrés and Coupland, 2012). How these environmental signals regulate flowering has been studied extensively in the Brassicaceae, and, in this family, the MADS-domain transcription factor FLOWERING LOCUS C (FLC) plays a central role in conferring a response to vernalization (Michaels and Amasino, 1999; Sheldon *et al.*, 1999). The transcriptional and post-transcriptional regulation of *FLC* and how these contribute to environmental responses have been reviewed in detail (Whittaker and Dean, 2017; Costa and Dean, 2019), but the downstream functions of FLC and how these provide further environmental and developmental check points on floral transition have received less attention. We discuss recent progress in defining the functions of FLC, how these integrate responses to daylength, temperature, and developmental age, and how they can confer developmental traits associated with divergence of annual and perennial life history.

FLC blocks floral transition by binding directly to genes encoding activators of flowering and repressing their transcription. During vernalization, *FLC* mRNA levels decrease and are stably repressed after vernalization, allowing target genes to be transcribed and flowering to occur (Michaels and Amasino, 1999; Sheldon *et al.*, 1999). The mechanisms by which *FLC* expression is stably repressed by cold have been recently reviewed (Whittaker and Dean, 2017; Costa and Dean, 2019), and a detailed description of these is outside the scope of this article. However, in general, three aspects of *FLC* regulation are critical in considering its effect on downstream targets. First, *FLC* repression occurs progressively during exposure to cold, and extended exposure of several weeks that is typical of winter is required for stable repression (Michaels and Amasino, 1999; Sheldon *et al.*, 1999). Exposure to cold for shorter periods can lead to partial repression of *FLC* and an incomplete flowering response (Coustham *et al.*, 2012; Lazaro *et al.*, 2018). Furthermore, the progressive repression of *FLC* across a tissue is due to a cell-autonomous mechanism in which *FLC* is fully, stably repressed in some cells but not at all in others (Angel *et al.*, 2011). The consequence of this progressive cell-autonomous repression of *FLC* activity for target gene activity probably varies for different genes and tissues. Secondly, stable *FLC* repression occurs through accumulation of modified histone 3 at the *FLC* gene. As the plant is exposed to cold, transcription of *FLC* is repressed and then trimethylation on lysine 27 of histone 3 (H3K27me3) accumulates at a nucleation point near the transcriptional start site, followed by spreading of the modification across *FLC* after return to warm temperatures (Finnegan and Dennis, 2007; Swiezewski *et al.*, 2009; Yang *et al.*, 2014). In Arabidopsis, the H3K27me3 mark persists on *FLC* after cold exposure has ended, resulting in the stable repression of *FLC* expression after cold exposure, although the duration of cold required for stable repression varies among accessions (Coustham *et al.*, 2012). In other Brassicaceae species, transcription of *FLC* orthologues is reactivated after vernalization (R. Wang *et al.*, 2009). These two contrasting patterns of *FLC* regulation have important consequences for the roles of downstream flowering genes and pathways (Hyun *et al.*, 2019). Thirdly, similar to most MADS-domain transcription factors (de Folter *et al.*, 2005), FLC binds DNA as heterodimers with other members of the family (Li *et al.*, 2008; Gu *et al.*, 2013) and, therefore, in considering its regulation of specific targets, it is important to assess the specificity of MADS-domain complexes that include FLC and the availability of partner proteins that might influence FLC function (Mateos *et al.*, 2015).

## Identification of FLC targets in leaves and apices

*FLC* is expressed broadly and therefore can potentially regulate targets in a wide range of tissues, including leaves, shoot apices, and root tips (Michaels and Amasino, 1999; Sheldon *et al.*, 2002; Bastow *et al.*, 2004). The significance of FLC activity in different tissues was supported by misexpression studies, which demonstrated that FLC can repress flowering when expressed in the phloem companion cells or the shoot meristem (Searle *et al.*, 2006). Furthermore, two major direct target genes that were identified early during the elucidation of Arabidopsis flowering pathways, *FLOWERING LOCUS T* (*FT*) and *SUPPRESSOR OF OVEREXPRESSION OF CONSTANS 1* (*SOC1*), are expressed in different tissues. *FT* encodes a RAF kinase inhibitor-like protein and is expressed in the vascular tissue of leaves, specifically in phloem companion cells (Kardailsky *et al.*, 1999; Kobayashi *et al.*, 1999; Chen *et al.*, 2018). It encodes a systemic flowering signal whose transcription is induced by inductive long-day photoperiods, and moves to the shoot apex, where it interacts with the bZIP transcription factor FD (Corbesier *et al.*, 2007; Jaeger and Wigge, 2007; Mathieu *et al.*, 2007; Abe *et al.*, 2019). FLC represses *FT* transcription by binding to *FT* chromatin, and specifically to the first intron of *FT* (Helliwell *et al.*, 2006; Searle *et al.*, 2006). In this way, FLC blocks expression of a critical component of the photoperiodic induction pathway. The second FLC target is *SOC1* (Hepworth *et al.*, 2002; Helliwell *et al.*, 2006; Searle *et al.*, 2006), which is expressed in leaves and the shoot apex (Samach *et al.*, 2000; Michaels *et al.*, 2005) and is one of the first genes induced by the FT signal at the shoot apex (Searle *et al.*, 2006). *SOC1* encodes a MADS-domain transcription factor that regulates several genes involved in floral transition at the shoot apex (Samach *et al.*, 2000; Immink *et al.*, 2012), and also contributes to floral transition in non-inductive short days (Moon *et al.*, 2003), when the FT pathway is not active. Thus, by repressing *SOC1*, FLC blocks expression of an early acting gene at the shoot meristem that contributes to several flowering pathways.

A broader understanding of FLC targets came from the genome-wide approach of ChIP sequencing (ChIP-seq). Applying this method, Deng *et al.* (2011) identified 505 target genes, including *FT* and *SOC1*. Plant MADS-domain proteins such as FLC bind to a specific CArG box DNA motif with the consensus sequence CC(A/T)_6_GG (Schwarz-Sommer *et al.*, 1992; Huang *et al.*, 1993). Consistent with this, the majority of genome-wide binding sites in promoter targets of FLC contained at least one consensus CArG-box motif CCAAAAAT(G/A)G with an AAA extension at the 3' end (Deng *et al.*, 2011). A later repetition of this experiment Mateos *et al.* (2015) identified 340 target genes with a 40% overlap with those identified by Deng *et al.* (2011), including many of the key flowering-related genes. The list of FLC targets included those active in pathways throughout development, although many were implicated in flowering (Table 1), including *TEMPRANILLO1* (Castillejo and Pelaz, 2008) and those encoding the AP2-domain transcription factors SCHLAFMÜTZE and TARGET OF EAT3 (Aukerman and Sakai, 2003; Schmid *et al.*, 2003), the *SQUAMOSA BINDING PROTEIN LIKE* (*SPL*) family member *SPL15*, which contributes to progression from the juvenile to the adult phase as well as floral transition (Schwarz *et al.*, 2008; Hyun *et al.*, 2016), and many genes involved in stress responses to cold, and light and hormone response pathways [jasmonic acid (JA), gibberellin (GA), ethylene, and auxin]. Additional targets include four genes involved in the circadian clock (*REVEILLE 2*, *FIONA 1*, *LATE ELONGATED HYPOCOTYL*, and *CONSTANS-LIKE1*), which might be relevant for the observation that FLC influences circadian period length (Salathia *et al.*, 2006). Comparison of the ChIP-seq list with genome-wide expression data comparing *FLC* and *flc* genotypes indicated that ~90% of the direct targets that were differentially expressed in *flc* mutants were increased in expression, supporting the idea that FLC acts mainly as a repressor of transcription (Mateos *et al.*, 2015).

**Table 1.** Validated direct gene targets of FLC/PEP1 involved in flowering time.

Gene	Target	Reference
FLC	*SUPPRESSOR OF OVEREXPRESSION OF CONSTANS1*	Helliwell *et al.* (2006)
		Hepworth *et al.* (2002)
		Searle *et al.* (2006)
FLC	*FT*	Deng *et al.* (2011)
		Searle *et al.* (2006)
FLC	*TARGET OF FLC AND SVP 1*	Richter *et al.* (2019)
FLC/PEP1	*SEPALLATA3*	Deng *et al.* (2011)
		Mateos *et al.*, 2017
FLC	*SQUAMOSA PROMOTER-BINDING-LIKE PROTEIN* 3	Deng *et al.* (2011)
FLC/PEP1	*SQUAMOSA PROMOTER-BINDING-LIKE PROTEIN 15*	Deng *et al.* (2011)
		Mateos *et al.* (2017)
PEP1	*SQUAMOSA PROMOTER-BINDING-LIKE PROTEIN* 8	Mateos *et al.* (2017)
FLC/PEP1	*SHORT VEGETATIVE PHASE*	Deng *et al.* (2011)
		Mateos *et al.*, 2017
FLC	*AGAMOUS-LIKE 16*	Deng *et al.* (2011)
FLC	*TEMPRANILLO 1*	Deng *et al.* (2011)
PEP1	*TARGET OF EAT 2*	Mateos *et al.* (2017)
FLC	*TARGET OF EAT 3*	Deng *et al.* (2011)
FLC	*SCHLAFMÜTZE*	Deng *et al.* (2011)
PEP1	*GIBBERELLIN 2-BETA-DIOXYGENASE 8*	Mateos *et al.* (2017)
PEP1	*GIBBERELLIN 3-BETA-DIOXYGENASE 2*	Mateos *et al.* (2017)
PEP1	*GIBBERELLIN RECEPTOR GID1B*	Mateos *et al.* (2017)

Helliwell *et al.* (2006) showed that FLC is part of a high molecular weight complex (600–800 kDa), suggesting that it might function as a tetramer and interact with other classes of proteins involved in transcriptional regulation, as described for other plant MADS-domain proteins (Theissen and Saedler, 2001; Smaczniak *et al.*, 2012). The SHORT VEGETATIVE PHASE (SVP) MADS-domain protein is another repressor of flowering in Arabidopsis (Hartmann *et al.*, 2000) and, similarly to *FLC*, decreases gradually in expression during floral transition (Gregis *et al.*, 2009; Jang *et al.*, 2009). The SVP and FLC proteins interact *in vivo*, and negatively regulate *SOC1* transcription by combinatorially binding to adjacent CArG-binding sites in the *SOC1* promoter (Li *et al.*, 2008). There is also a striking overlap between FLC and SVP target genes genome-wide (Tao *et al.*, 2012; Gregis *et al.*, 2013; Mateos *et al.*, 2015). In direct comparisons, FLC and SVP bound to 183 common genes, often to the same genomic regions within those genes; however, some genes were exclusively regulated by binding of one of the proteins (Mateos *et al.*, 2015). Furthermore, a notable difference was that FLC regulated twice as many genes in leaves as SVP, and SVP had a more significant effect on gene expression in the apex than FLC. Mutation of *SVP* did not completely suppress the delay in flowering caused by FLC, suggesting that FLC might interact with other proteins or act alone. In agreement with this conclusion, FLC also physically interacts with the related floral repressor proteins MADS AFFECTING FLOWERING 3 (MAF3), MAF4, and FLOWERING LOCUS M (FLM) (de Folter *et al.*, 2005; Gu *et al.*, 2013; Posé *et al*., 2013), and these interactions are required for full floral repression by FLC (Gu *et al.*, 2013). Furthermore, in addition to the CArG-box motif found at peaks surrounding FLC-binding sites in the ChIP-seq data, the G-box motif, CACGTG (Deng *et al.*, 2011; Mateos *et al.*, 2015), was enriched, suggesting possible combinatorial interactions at target genes between FLC and unrelated transcription factors.

In addition to *A. thaliana*, *FLC* orthologues repress flowering and contribute to vernalization response in many other species from different Brassicaceae clades, including *Arabidopsis lyrata* (Kemi *et al.*, 2013), *Arabis alpina* (R. Wang *et al.*, 2009; Albani *et al.*, 2012), *Arabidopsis halleri* (Aikawa *et al.*, 2010; Nishio *et al.*, 2016), *Arabidopsis arenosa* (Baduel *et al.*, 2016), *Capsella rubella* (Guo *et al.*, 2012), *Cardamine hirsuta* (Cartolano *et al.*, 2015), *Brassica rapa* (Schranz *et al.*, 2002), and *Boechera stricta* (Lee *et al.*, 2018). However, the relatedness of their target genes in these different species has not been extensively studied. Comparative ChIP-seq analysis of FLC in *A. thaliana* and its orthologue PERPETUAL FLOWERING 1 (PEP1) in *A. alpina* identified a conserved group of 39 target genes, and these included genes with important functions in floral transition, such as *SPL15*, *SOC1*, and *SEP3* (Mateos *et al.*, 2017). However, overall, only 14% of binding sites were conserved. In addition, FLC and PEP1 also regulate common pathways related to GA and cold response, but mostly by binding to different genes, and many of these binding sites were specific to the *A. alpina* lineage, suggesting that the regulation of these pathways has been evolutionarily recruited independently following speciation and might represent convergent evolution (Mateos *et al.*, 2017; Tilmes *et al.*, 2019). The involvement of FLC in regulating GA responses was also supported by demonstration of an *in vivo* interaction between the C-terminus of the MADS domain of FLC and the N-terminal leucine heptad repeat I (LHRI) domain of DELLA proteins such as RGA in Arabidopsis (Li *et al.*, 2016). These DELLA proteins are important mediators of GA signalling that are degraded in the presence of GA (Schwechheimer, 2012). Because DELLA proteins are believed not to bind DNA directly, their association with flowering time genes might in part be mediated by FLC (Li *et al.*, 2016).

## The FLC target *SPL15* is regulated by miR156 to confer age-dependent responses to vernalization

**Fig. 1.**
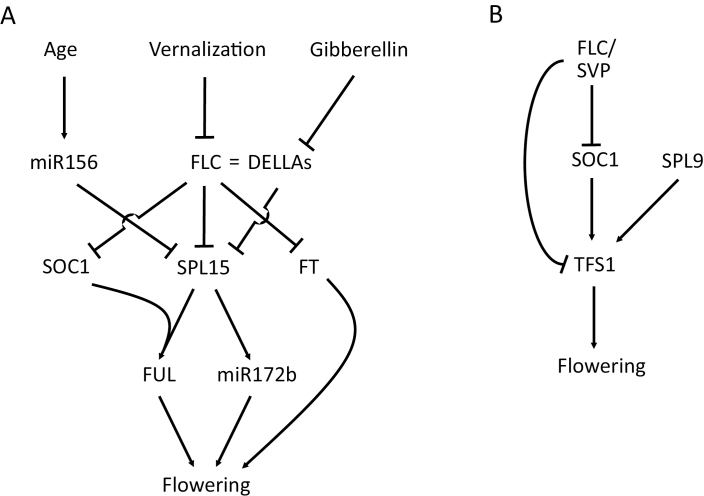
Schematic representation of the gene regulatory network controlled by FLC. (A) FLC is a master regulator of flowering that integrates cues from several flowering pathways in Arabidopsis. *FLC* expression is repressed by the cold-induced vernalization pathway. FLC directly represses the florigen-encoding gene *FT* and *SQUAMOSA PROMOTER BINDING-LIKE PROTEIN 15* (*SPL15*). SPL15 is additionally post-translationally regulated by the gibberellin pathway via DELLA proteins and at the post-transcriptional level by the ageing pathway via miR156. The MADS-domain protein SUPPRESSOR OF OVEREXPRESSION OF CONSTANS1 (SOC1) is encoded by another direct target of FLC, and cooperates with SPL15 to activate target genes such as *FRUITFULL* and *MIR172B*. (B) A type II coherent feed-forward loop regulates expression of the positive floral regulator *TARGET OF FLC AND SVP1* (*TFS1*). FLC and SVP directly repress *TFS1* transcription and that of its positive activator *SOC1*. Activation of *TFS1* expression in the inflorescence meristem also involves SPL9.

One of the targets of FLC, *SPL15*, represents a convergence point of flowering pathways regulated by vernalization, the age of the plant, and GA. In Arabidopsis, SPL15 and a further 10 members of the SPL family of transcription factors are negatively regulated at the post-transcriptional level by miRNA156 (miR156) and closely related miR157 (Rhoades *et al.*, 2002; Guo *et al.*, 2008). These miRNAs are expressed at high levels in the cotyledons and leaves produced early in shoot development, and progressively decrease in abundance in leaves formed later on the shoot (Wu and Poethig, 2006; Yang *et al.*, 2013; Yu *et al.*, 2013). During vegetative development, these miRNAs repress the transition from the juvenile to adult phase, so that their progressive reduction during shoot development allows the acquisition of adult traits such as abaxial trichomes on leaves (Wu *et al.*, 2009). The abundance of miR156 also falls in shoot apices, where it controls floral transition (J.W. Wang *et al.*, 2009; Bergonzi *et al.*, 2013). Overexpression of *MIR156f* in the shoot apical meristem (SAM) delayed floral transition (J.W. Wang *et al.*, 2009). Several SPL transcription factors that contain miR156 target sequences in their cognate mRNA are expressed in the SAM, including SPL3, SPL4, SPL5, SPL9, and SPL15 (Cardon *et al.*, 1997; J.W. Wang *et al.*, 2009; Yamaguchi *et al.*, 2009; Hyun *et al.*, 2016). Among these, loss-of-function mutant alleles of *SPL15* delay flowering under short days (SDs) (Hyun *et al.*, 2016; Xu *et al.*, 2016). Furthermore, mutations in the miR156 recognition sequence of *SPL15* (*rSPL15*) increase the abundance of SPL15 and confer early flowering (Hyun *et al.*, 2016). In wild-type plants, SPL15 accumulates in the SAM under SDs prior to floral induction, and rSPL15 accumulates earlier (Hyun *et al.*, 2016), correlating with earlier flowering. Taken together, these results indicate that the FLC target *SPL15* promotes flowering under SDs and its ability to promote flowering is repressed in younger plants by miR156 (Fig. 1A). In contrast to its late-flowering phenotype under SDs, the *spl15* mutant is not late flowering under long days (LDs). This conditionality of the phenotype dependent on daylength was proposed to be due to the photoperiodic flowering pathway bypassing the requirement for SPL15 under LDs. This model was confirmed genetically by combining the *ft* and *twin sister of ft* (*tsf*) mutations, which block the photoperiodic pathway, with the *spl15* mutant (Hyun *et al.*, 2019). The triple mutant *spl15 ft tsf* flowered much later than *ft tsf* under LDs. Thus, *spl15* exhibits a conditional effect on flowering time, only being required under conditions in which the photoperiodic pathway is not active.

**Fig. 2.**
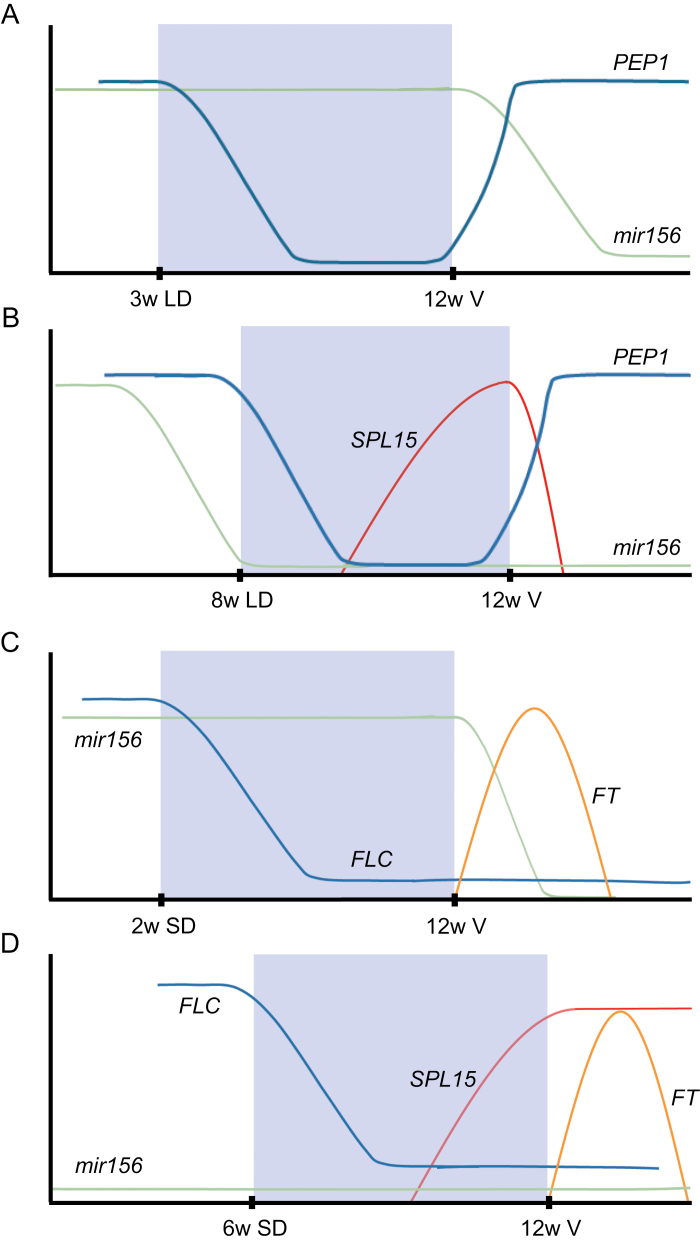
FLC and its orthologues in other species regulate flowering in response to vernalization by controlling two major floral-promoting pathways involving SPL15 and FT. In perennial *Arabis alpina* plants exposed to vernalization, *FT* expression is repressed by the FLC orthologue PEP1 both before and after vernalization, when PEP1 is reactivated by warm conditions (A and B). Therefore, flowering is dependent on the miR156/SPL15 module. When young meristems are vernalized, miR156 levels are high and repress floral transition by maintaining low *SPL15* levels, so no flowering occurs (A). Only older meristems can flower during the vernalization period when the level of miR156 has fallen and SPL15 mRNA can increase during vernalization (B). In the winter-annual *Arabidopsis thaliana*, *FLC* is transcriptionally repressed during vernalization in short days in the cold and remains stably repressed in subsequent warm temperatures (C and D). This allows plants to flower by activation of *FT* transcription after cold treatment via the photoperiodic pathway irrespective of age, even when miRNA156 levels are high in young plants that are vernalized (C). Stable repression of FLC transcription in annuals therefore allows plants to flower independently of age through the FT pathway after vernalization, thus bypassing dependency on the miRNA156/SPL15 module. The graphs (A–D) depict the relative level of *FLC*, *PEP1*, *SPL15*, and *FT* mRNAs, and miRNA156 on the *y*-axis. V, vernalization; w, weeks; SD. short days; LD, long days. The blue box represents a vernalization treatment.

The functional importance of SPL15 and miR156 in vernalization response downstream of FLC first became evident from analysis of the perennial Brassicaceae species *A. alpina* and *Cardamine flexuosa* (Bergonzi *et al.*, 2013; Zhou *et al.*, 2013). These plants do not flower if exposed to vernalization soon after germination, but do when exposed to vernalization when 4–5 weeks old. This dependency on the age of the plant can be defined as the acquisition of competence to flower in response to vernalization. It cannot be explained by age-dependent reduction in *FLC* mRNA, because exposure of young plants to vernalization caused repression of *FLC* transcription (Bergonzi *et al.*, 2013; Zhou *et al.*, 2013). However, because the ability to respond to vernalization correlated with miR156 reaching trough levels, the progressive reduction in miR156 level in the shoot apex was proposed to confer age-dependent responses to vernalization. Consistent with this, transgenic plants that expressed *MIR156f* from the viral *35S* promoter never flowered in response to vernalization, and a reduction in miR156 activity by expressing a miR156 mimic that sequesters miR156 enabled younger plants to flower in response to vernalization (Bergonzi *et al.*, 2013; Zhou *et al.*, 2013). This effect of miR156 in *A. alpina* was later shown to depend upon the orthologue of *SPL15* (*AaSPL15*) (Hyun *et al.*, 2019). Loss-of-function mutations in *AaSPL15* prevented flowering in response to vernalization, as observed for *MIR156f*-overexpressing plants; furthermore, *rSPL15* plants acquired competence to respond to vernalization earlier than the wild type. Notably, age was measured independently in the SAM and each axillary meristem, suggesting that the mechanism of miR156 down-regulation by age is regulated autonomously in different meristems of the same plant (Park *et al.*, 2017; Hyun *et al.*, 2019). Therefore, an age-dependent vernalization response can be explained by repression of *SPL15* transcription by FLC and *SPL15* post-transcriptional repression by miR156 in individual meristems, so that this double lock on *SPL15* expression is only relieved when *FLC* expression is reduced during vernalization and miR156 levels decrease in older meristems (Fig. 2).

Finally, SPL15 is negatively regulated at the post-translational level by GA. SPL15 interacts with the DELLA protein RGA in yeast and *in planta*, and reduction in GA levels in the shoot meristem increases the abundance of RGA and the amount of the RGA–SPL15 complex (Hyun *et al.*, 2016). The interaction between RGA and SPL15 was proposed to reduce SPL15 activity, because lowering GA levels at the shoot apex by expression of a GA catabolic enzyme reduced the ability of rSPL15 to promote early flowering. DELLA proteins also interact with SPL9 (Yu *et al.*, 2012; Yamaguchi *et al.*, 2014), suggesting that the regulation of the activity of SPL transcription factors by GA during reproductive development is more prevalent (Zhang *et al.*, 2007). It is relevant that FLC and its orthologue in *A. alpina*, PEP1, directly bind to several genes that encode enzymes required for GA biosynthesis or for GA signalling (Deng *et al.*, 2011; Mateos *et al.*, 2015; Tilmes *et al.*, 2019). The transcription of some of these genes also increases during vernalization (Tilmes *et al.*, 2019). This observation suggests that FLC ensures that SPL15 is inactive prior to vernalization by effectively regulating its expression via a feed-forward loop: first, FLC binds directly to the *SPL15* promoter to repress its transcription; and, secondly, FLC reduces GA biosynthesis, which increases the level of DELLA proteins that inhibit SPL15 activity. SPL15 expression is then activated transcriptionally and post-translationally during vernalization as *FLC* transcription is repressed. Because GA levels increase at about the time of flowering in apices of plants grown under SDs (Eriksson *et al.*, 2006), this post-translational regulation of SPL15 probably also contributes to its appropriate temporal activation under non-inductive conditions.

## *FT* and *SPL15* are genetically redundant FLC targets that determine whether flowering occurs during or after cold exposure

Expression of *FLC* prior to vernalization blocks floral induction by directly repressing the transcription of a set of flowering genes that are described above, whereas the transcriptional repression of *FLC* by cold during vernalization allows these targets to be expressed and floral induction to proceed. In *A. thaliana*, floral induction is usually considered to occur after vernalization, when plants in which *FLC* transcription is repressed are exposed to LDs that mimic spring conditions and allow activation of the photoperiodic pathway (Coustham *et al.*, 2012) (Fig. 2). During the vernalization period, when plants are exposed to cold, the photoperiodic pathway is not active, because vernalization typically occurs under SDs or low light, analogous to winter conditions. Thus, stable repression of *FLC* transcription renders *A. thaliana* competent to flower through the photoperiodic pathway after vernalization. The mobile florigen protein FT is a component of the photoperiodic pathway and is transcribed only in LDs following vernalization. Furthermore, as *FT* transcription is directly repressed by FLC, the photoperiodic flowering response after vernalization depends upon stable repression of *FLC* transcription by cold. Thus, *A. thaliana*, and other species in which *FLC* transcription is stably repressed by vernalization, can undergo floral induction after vernalization through this FT-based route. As described above, because FT can also bypass the requirement for the miR156/SPL15 module, stable repression of *FLC* transcription allows floral induction to occur after vernalization, independently of the age of the plant. Genetic support for this model came recently from comparing the vernalization response of *A. thaliana FRI FLC ft tsf* and *FRI FLC* plants (Hyun *et al.*, 2019). Young *FRI FLC ft tsf* plants, in which miR156 levels were still high, flowered much later after vernalization than *FRI FLC* plants, whereas older *FRI FLC ft tsf* plants, in which miR156 levels had declined, flowered at the same time as *FRI FLC* when exposed to vernalization.

In contrast, transcription of *FLC* orthologues in perennial Brassicaceae species, such as *PEP1* of *A. alpina*, is reactivated after vernalization (R. Wang *et al.*, 2009; Baduel *et al.*, 2016; Kiefer *et al.*, 2017) and, therefore, PEP1 represses transcription of *FT* when *A. alpina* is exposed to LDs after vernalization (Hyun *et al.*, 2019) (Fig. 2). Consequently, flowering in *A. alpina* is initiated during cold exposure when *PEP1* transcription is repressed. Because the photoperiodic pathway is effectively blocked by PEP1 before and after vernalization, these plants are dependent upon the miR156/*SPL15* module for floral induction during vernalization, which explains why they can only respond to vernalization when meristems reach the age at which miR156 levels have fallen. The importance of reactivation of *PEP1* after vernalization in conferring an age-dependent vernalization response in *A. alpina* was recently supported by genetic data. *Arabis alpina pep1* mutants carrying a stably repressed allele of *FLC* from annual *Arabis montbretiana* could respond to vernalization as young plants, as previously shown for *A. thaliana* (Hyun *et al.*, 2019).

Thus, by negatively regulating both *SPL15* and *FT*, FLC and its orthologues control both major pathways that promote flowering in response to vernalization. Stable repression of *FLC* transcription in annuals allows plants to flower independently of age through the FT pathway after vernalization, whereas reactivation of *FLC* orthologues in perennials blocks *FT* transcription, which forces these plants to flower through the age-dependent SPL15 pathway. Recently, winter varieties of *Brassica napus* that flower in response to vernalization were found to produce flower buds in late autumn and open flowers in spring (O’ Neill *et al.*, 2019). In these plants, floral induction occurs under vernalizing conditions in autumn when the photoperiodic pathway is not expected to be active, and therefore might initiate flowering via the miR156/SPL15 pathway. This possibility extends the previously noted similarities between flowering of *B. napus* crops and perennials such as *A. alpina* (O’ Neill *et al.*, 2019), and is significant for breeding for flowering time in winter crops.

## A regulatory module involving MADS-domain and B3-type transcription factors controlled by FLC at the shoot meristem

A further direct target of FLC that was recently shown to contribute to a feed-forward loop in the shoot meristem is *TARGET OF FLC AND SVP 1* (*TFS1*), which encodes a B3-type transcription factor that promotes flowering (Richter *et al.*, 2019). *TFS1* was identified by mining genome-wide ChIP-seq and RNA-seq data as a gene specifically expressed in shoot apices and directly bound by FLC and its interacting partner SVP (Mateos *et al.*, 2015; Richter *et al.*, 2019). The gene is expressed on the flanks of the shoot meristem specifically during and after floral transition, and *tfs1* mutants are late flowering. In *FRI FLC* plants, the mRNA level of *TFS1* increases during vernalization as *FLC* expression falls. Transcriptional activation of *TFS1* in this precise temporal and spatial pattern involves SPL9, the paralogue of SPL15, and the MADS-domain transcription factor SOC1 (Richter *et al.*, 2019) (Fig. 1B). SOC1 and SPL9 physically interact and both bind directly to different sites within the *TFS1* promoter, suggesting that their interaction might induce looping at the locus that is required to activate *TFS1* transcription. *SOC1* is not only a well-established target of FLC, whose transcription is repressed by direct binding of FLC to its promoter and activated during vernalization when *FLC* transcription is repressed (Hepworth *et al.*, 2002; Searle *et al.*, 2006; Deng *et al.*, 2011), but is also one of the earliest acting transcription factors during floral transition (Samach *et al.*, 2000; Immink *et al.*, 2012). Thus, FLC represses *TFS1* transcription both by direct binding to its promoter and by repressing transcription of its major direct upstream regulator SOC1, creating a coherent type-2 feed-forward loop (Alon, 2007). Such a loop might delay the expression of *TFS1* rather than inducing a more rapid response to FLC down-regulation during vernalization (Mangan and Alon, 2003).

## Mechanism by which FLC represses transcription of targets

The predominant genome-wide function of FLC is as a repressor of transcription (Mateos *et al.*, 2015), but the mechanism by which this occurs has not been elucidated in detail, although it probably involves protein partners. FLC is a type II MADS-domain protein that possesses multiple domains that can potentially interact with other proteins. These include the keratin-like domain, also known as the K-box, the intervening domain, the MADS-domain that binds DNA, and the C-terminal domain that can stabilize protein complexes and regulate transcription (Kaufmann *et al.*, 2005). Thus, the target specificity of FLC and efficacy of its repression function is potentially conferred by its interaction partners, or the cofactors of the MADS-domain proteins with which it heterodimerizes (Li *et al.*, 2008; Gu *et al.*, 2013).

Several proteins that interact with FLC are implicated in chromatin modifications: FLC physically interacts with EMBRYONIC FLOWER 1 (EMF1) and is required for FLC-mediated *FT* repression (Wang *et al.*, 2014). EMF1, LIKE HETEROCHROMATIN PROTEIN 1 (LHP1), and the H3K4me3 demethylase JMJ14/PKDM7B form a polycomb group protein complex termed EMF1c, which is a polycomb repressive complex1 (PRC1)-like complex that represses *FT* transcription (Wang *et al.*, 2014). EMF1 is also associated with many genomic sites, including FT chromatin, which are marked with H3K27me3 deposited by PRC2 (Kim *et al.*, 2012). This involvement of FLC with polycomb repressive complexes suggests that PcG-mediated gene silencing contributes to repression of gene expression by FLC. This was supported for the direct target of FLC and SVP, *TFS1*, which was found to possess a high level of repressive H3K27me3 marks at sites that were also occupied by LHP1 and were dependent on FLC and SVP (Richter *et al.*, 2019). The level of H3K4me3 is enriched at the chromatin of actively transcribed genes, and the level of this mark was low at the *TFS1* locus in the presence of FLC and SVP, and was enriched in single and double mutants of *flc* and *svp* (Richter *et al.*, 2019). Therefore, the presence of repressive and permissive chromatin modifications at *TFS1* is consistent with the known antagonistic dynamics of chromatin marks during floral transition (Engelhorn *et al.*, 2017).

Transcription of *TFS1* is repressed by binding of FLC and SVP 3' to the *TFS1* stop codon and their interaction with a PRC complex and LHP1 that associate with the gene body of *TFS1*, suggesting that the resulting complex forms a chromosomal loop. The formation of this ‘locked’ loop is dependent on CURLY LEAF (CLF), FLC, or SVP (Richter *et al.*, 2019). Therefore, the transcriptional repression of *TFS1* by FLC and SVP consists of two component processes: the formation of a chromatin loop that requires FLC; and SVP binding at the 3' end of the gene and high levels of H3K27me3 within the gene body. SOC1 also activates *TFS1* transcription by binding to the 3' end of *TFS1* and reducing SVP recruitment to the same region (Richter *et al.*, 2019). It remains unclear how general the mechanisms of action of FLC on *FT* and *TFS1* are for other targets. However, based on *TFS1* regulation, antagonistic chromatin remodelling via FLC and other MADS-domain transcription factors appears to represent the central mechanism that defines the spatiotemporal expression of downstream targets.

## Quantitative effects of FLC on target genes and its consequences for floral transition and inflorescence development

The level of *FLC* mRNA varies tremendously among Arabidopsis accessions and is correlated with flowering time (Michaels *et al.*, 2003; Lempe *et al.*, 2005; Shindo *et al.*, 2005; Sasaki *et al.*, 2018). In a genome-wide expression analysis of 132 accessions, a subset of 38 genes whose expression was correlated with flowering time included *FLC* and three downstream targets, *FT*, *SOC1*, and *SPL15* (Sasaki *et al.*, 2018). Thus, increasing levels of *FLC* mRNA can lead to later flowering phenotypes, presumably by reducing the expression of known target genes. Variation in the *FLC* expression level is largely due to allelic variation either at *FLC* or at the upstream *FRIGIDA* (*FRI*) locus. Natural allelic variation in *FRI* has been analysed for >1000 Arabidopsis accessions (Zhang and Jimenez-Gomez, 2020), and has been estimated to account for ~70% of diversity in flowering time (Shindo *et al.*, 2005). FRI is recruited to the *FLC* locus as part of a large protein complex containing chromatin modifiers to increase *FLC* transcription (Li *et al.*, 2018). Many early-flowering summer-annual Arabidopsis accessions arose from winter-annual progenitors by the loss of *FRI* function (Johanson *et al.*, 2000). Other early-flowering accessions possess *FLC* alleles that are expressed weakly (Michaels *et al.*, 2003) or in which the duration of vernalization required for stable repression varies (Coustham *et al.*, 2012) due to *cis*-acting variation. Among 173 natural Swedish accessions, enormous variation for flowering time at growth temperatures of 10°C and 16°C was detected, and genome-wide association analysis identified a peak on the promoter of the *FLC* gene (Sasaki *et al.*, 2015). Variation in the expression of *FLC* orthologues is probably also important in determining flowering time in other Brassicaceae species but, as many are allopolyploids containing several copies of different *FLC* orthologues, the combined effects of these on flowering time and vernalization response can be complex (Schiessl *et al.*, 2019; Takada *et al.*, 2019).

In addition to affecting flowering time in the absence of vernalization treatment, allelic heterogeneity at the *FLC* locus can determine the duration or temperature of vernalization required to induce flowering. Accessions differ in the duration of vernalization required for stable repression of *FLC*, and therefore for activation of the *FT* target gene after vernalization (Coustham *et al.*, 2012). The coding sequence of FLC is remarkably conserved among Arabidopsis accessions (Li *et al.*, 2014) and most polymorphisms at the *FLC* locus are associated with *cis*-acting variation in *FLC* non-coding regions. Some of these, for example in the lov-1 accession, are near the transcriptional start site, close to the site at which the H3K27me3 mark first increases during vernalization (Coustham *et al.*, 2012). The lov-1 plants require exposure to cold for longer than is necessary for full vernalization of other *A. thaliana* accessions and, after shorter vernalization periods, *FLC* expression in lov-1 is reactivated in individual cells in subsequent warm conditions (Coustham *et al.*, 2012), a feature that is characteristic of perennial Brassicaceae species. A combination of *cis*-acting single nucleotide polymorphisms (SNPs) at lov-1 *FLC* quantitatively mediates instability of *FLC* repression by disrupting stable long-term chromatin silencing of the locus, probably via histone modification feedback (Qüesta *et al*., 2020). Another study tested the responses of 47 Arabidopsis accessions containing the major haplotypes of *FLC* to short vernalization treatments of 4 weeks, and detected variation in the ability to stably repress *FLC* transcription (Li *et al.*, 2014). Variation of these haplotypes in this response was again associated with non-coding sequence variation presumably linked to the histone modifications at *FLC* related to stable repression (Li *et al.*, 2014). Differences in *FLC* expression patterns due to variations in chromatin silencing between annual and perennial Brassicaceae depend on *cis*-polymorphisms in non-coding FLC sequences (Kiefer *et al.*, 2017), and might therefore represent a general mechanism for life history evolution. Thus, accessions vary in the duration of vernalization required to stably repress *FLC*, and allow the promotion of flowering through activation of *FT*, and presumably other targets. Furthermore, the duration of cold treatment required for full vernalization is likely to be more complex in the fluctuating conditions that prevail during natural growth (Burghardt *et al.*, 2016). In addition to affecting floral transition, partial vernalization treatments can affect the extent of inflorescence development. This was most clearly demonstrated in *A. alpina*, where incomplete vernalization led to reactivation of the *FLC* orthologue *PEP1* in the inflorescence, causing floral reversion and reduced inflorescence size (Lazaro *et al.*, 2018). Similarly, in *A. thaliana*, high levels of *FLC* expression caused by allelic variation at the upstream regulator *ENHANCER of AG-4 2* (*HUA2*) can also cause floral reversion phenotypes in the inflorescence and vegetative rosettes to form within the inflorescence (Wang *et al.*, 2007). These phenotypes, which are probably caused by repression of FLC targets during inflorescence development, may also persist after incomplete vernalization.

The stable repression of *FLC* by vernalization occurs cell-autonomously via an ‘all or nothing’ bistable state (Angel *et al.*, 2011). Therefore, incomplete vernalization would be expected to result in tissues in which *FLC* is completely switched off in some cells, but is still expressed at pre-vernalization levels in others (Angel *et al.*, 2011). How this would affect the expression of target genes in different tissues is unknown. However, at least for *SOC1*, a gradual increase that was the reciprocal of the effect on *FLC* transcription was observed during vernalization (Sheldon *et al.*, 2006), suggesting that *SOC1* may be activated in a cell-autonomous manner, although regulation of *SOC1* is complicated by an FLC-independent acute induction by cold (Sheldon *et al.*, 2006). Activation of *FT* transcription in a cell-autonomous manner may be sufficient to activate floral induction, because the FT protein induces flowering cell non-autonomously (Corbesier *et al.*, 2007). However, for targets encoding transcription factors such as SOC1, SPL15, SEP3, or TFS1, it is unclear that they could confer inflorescence and floral meristem identity cell non-autonomously. Thus, partial vernalization treatments would be expected to have complex and unpredictable effects on inflorescence development.

## Perspectives

Only a subset of FLC target genes has been incorporated into gene regulatory networks that control floral transition. Notably, those studied in detail encode transcription factors (e.g. SOC1, SPL15, and TFS1) or components of transcriptional complexes (e.g. FT), whereas genes encoding other classes of protein, including enzymes of unknown function or involved in phytohormone biosynthesis, remain to be studied in detail. Established target genes of FLC, such as *FT* and *SPL15*, define key pathways that regulate the floral transition in different environments or physiological contexts. These pathways intersect with the patterns of transcriptional repression of *FLC* or its orthologues in particular species to confer different flowering behaviours. For example, the unstable repression of the *FLC* orthologue *PEP1* in *A. alpina* forces this species to flower through the SPL15 pathway, which is also controlled by miR156, generating an age-based check point on vernalization response (Hyun *et al.*, 2019) (Fig. 2). Other such check points may exist and explain how, in some contexts, *FLC* repression and floral induction can occur during vernalization and in autumn, but that flowers and inflorescences only fully develop in spring (Kemi *et al.*, 2019; O’ Neill *et al.*, 2019). Determining the spatial and temporal patterns of expression of further FLC targets and the regulatory networks they control during vernalization may contribute to defining other check points at which flowering can be environmentally regulated downstream of FLC. Comparative approaches among species are likely to be essential to understand the full role of FLC in flowering processes (Mateos *et al.*, 2017; Hyun *et al.*, 2019; Kemi *et al.*, 2019; O’ Neill *et al.*, 2019), as the flowering behaviour of *A. thaliana* is highly derived and dominated by the FT pathway. Similarly, further studies under natural conditions will define how the networks controlled by FLC contribute to seasonal patterns in ecologically relevant conditions (Burghardt *et al.*, 2016; O’ Neill *et al.*, 2019).
